# Role of signal transduction and microRNAs on the immunogenicity of melanoma cells

**DOI:** 10.1186/1479-5876-13-S1-K15

**Published:** 2015-01-15

**Authors:** Stefanie Meyer, Diana Handke, Anja Mueller, Anne Meinhardt, Simon Jasinski-Bergner, Stephan Mages, Juergen Bukur, Marco Donia, PerThor Straten, Barbara Seliger

**Affiliations:** 1 University Hospital Regensburg, 93053 Regensburg, Germany; 2 Institute of Medical Immunology, Martin Luther University Halle-Wittenberg, 06112 Halle, Germany; 3 University Hospital Herlev, 2730 Herlev, Denmark; 4 TBC

## 

Melanoma tumors are heterogeneous and involve different processes generating cells with variable metastatic capacity, which also results in distinct clinical outcome of patients and variations in therapy responses. This might be due to different strategies leading to evasion of T and or NK cell-mediated surveillance, which is accompanied by disease progression and a poor survival of melanoma patients. Therefore, the characterization of immune escape mechanisms might contribute to a better understanding of the development of the aggressive melanoma phenotype. This study analysed the underlying molecular mechanisms of HLA class I abnormalities and aberrant HLA-G expression in melanoma. A reduced mRNA and/or protein expression of various components of the MHC class I antigen processing machinery (APM) was identified in a large series of melanoma cell lines and lesions, which could be correlated with an increased tumor grading. In addition, adoptive T cell therapy also caused an escape from immune cell recognition by down-regulation components of the antigen processing machinery (APM). Recently, microRNAs (miRs) targeting HLA class I APM components were identified suggesting an important role for posttranscriptional control in this process. These miRs have functional activities on APM components, thereby also affecting HLA class I surface expression. Thus, these miRs might be used as novel targets for the treatment of melanoma or for selection of melanoma patients undergoing the most effective (immuno)therapy. In addition, a direct link of the IFN signalling pathway and the constitutive HLA class I APM component expression was found in melanoma. Down-regulation or loss of single components of the IFN-γ signal cascade was detected, which could be due to structural alterations, epigenetic mechanisms, such as methylation and/or histone deacetylation, or transcriptional/post-transcriptional deregulation. This was accompanied by a heterogeneous response of melanoma cells to IFN-γ treatment. Alterations in the IFN-γ signalling pathway were directly associated with a down-regulation of constitutive HLA class I components as demonstrated in melanoma lesions as well as in melanoma cell lines. Using shRNA-mediated silencing a direct effect of STAT1, JAK1 and JAK2 on MHC class I surface expression was confirmed. These data may provide novel insights into the complex regulation of HLA class I expression in melanoma and gives a rational for excluding patients for specific immunotherapies. Furthermore, strategies will be developed for restoration of the HLA class I positive phenotype.

